# Exosomes-Transferred lncRNA H19 Reverses Osimertinib Resistance by Upregulating PTEN via Sponging miR-148-3p in Non-Small Cell Lung Cancer

**DOI:** 10.32604/or.2026.078665

**Published:** 2026-05-21

**Authors:** Weixiang Song, Yanbo Zhang, Xubo Shen, Qin Yu, Yujin Liu, Yongchun Yu, Rui Chen

**Affiliations:** 1 Department of Oncology, Yueyang Hospital of Integrated Traditional Chinese and Western Medicine, Shanghai University of Traditional Chinese Medicine, Shanghai, China; 2 Department of Oncology, Shuguang Hospital Shanghai University of Traditional Chinese Medicine, Shanghai, China; 3 Institute for Thoracic Oncology, Shanghai Chest Hospital, Shanghai Jiao Tong University, Shanghai, China

**Keywords:** lncRNA H19, phosphatase and tensin homolog (PTEN), exosomes, osimertinib, non-small cell lung cancer

## Abstract

**Objective:** Osimertinib can selectively inhibit both epidermal growth factor receptor (EGFR) sensitizing and T790M gatekeeper mutations, and has shown remarkable therapeutic effects in patients with lung adenocarcinoma. However, almost all patients inevitably develop drug resistance. Herein, we sought to **clarify** the roles of exosomal lncRNA H19 in modulating osimertinib resistance, focusing on the PI3K-PTEN-Akt signaling axis. **Methods:** Functional assays, including cell viability assay, colony formation, cell apoptosis and xenograft mouse, employed in evaluate the effects of exosomal lncRNA H19 on cell growth and apoptosis. RNA quantitation and western blot were adopted to demonstrate the regulatory roles of exosomal lncRNA H19 in PI3K-PTEN-Akt signaling pathway. Immunofluorescence was applied to obverse the function and distribution of exosomes. Furthermore, dual-luciferase reporter analysis combined with RNA immunoprecipitation (RIP) was applied to verify the molecular interaction between lncRNA H19 and phosphatase and tensin homolog (PTEN). **Results:** LncRNA H19 exhibited obviously decreased expression in H1975R cells and their secreted exosomes. Overexpression of H19 enhances the cytotoxicity of osimertinib, inhibits the growth of H1975R cells, and promotes apoptosis. Conversely, H19 silencing promotes osimertinib resistance in H1975 cells and enhances the cell-resistant phenotype. Furthermore, exosome-transferred lncRNA H19 sponged miR-148-3p to augment PTEN expression, which in turn inactivated the PI3K-Akt signaling pathway and ultimately induced cell apoptosis. **Conclusion:** Exosome-encapsulated lncRNA H19 can be delivered to osimertinib-resistant H1975R cells, thereby reversing resistance through the miR-148-3p/PTEN/PI3K-Akt axis. Our results uncover a potential therapeutic approach to surmount osimertinib resistance in lung cancer.

## 1 Introduction

Globally, lung cancer ranks among the predominant malignancies and is the predominant cause of cancer-related mortality, amounting to roughly 2.5 million new diagnoses cases and 1.8 million mortalities in 2022 [1]. In patients with *EGFR*-mutated non-small cell lung cancer (NSCLC), EGFR tyrosine kinase inhibitors (TKIs) are routinely administered as first-choice therapy [2]. EGFR mutations happen in 30–50% of patients with pulmonary adenocarcinoma in Asian [3]. More than 80% of EGFR gene mutations reside in exon 19 (del746_A750) or exon 21 (L858R), and tumors have a high response to EGFR-TKIs, for instance, gefitinib, erlotinib, and afatinib, the primary treatment with these drugs displayed objective response rates of 66.9–73.7%, and progression free survival of 10.8–13.1 months, both of which were superior to those with platinum-based chemotherapy [4,5,6]. Despite high tumor response rates, most patients develop progressive disease within 1–2 years after administration of first-generation EGFR-TKIs [7]. About 60% of patients have a Thr790Met mutation (T790M) in EGFR gene, which leads to resistance to EGFR-TKIs [8].

Osimertinib, a third-generation orally administered non-reversible EGFR-TKI, suppresses sensitizing and T790M EGFR mutation [9]. The AURA3 trial (NCT02151981) indicated the osimertinib was superior to platinum plus pemetrexed therapy, the objective response rates (ORR) of osimertinib in T790M-positive lung cancer patients was 71% (vs. 31%), the media progression free survival (PFS) was 10.1 months (vs. 4.4 months) [10], and the median overall survival (OS) was 26.8 months (vs. 22.5 months) [11]. Nevertheless, disease progression and secondary resistance to osimertinib inevitably occur in almost all patients. When osimertinib develops resistance, EGFR is activated and triggers downstream PI3K-Akt and MAPK signaling pathways, leading to cell growth, invasion and distant spread [12]. The PI3K-Akt pathway is negatively modulated via the tumor suppressor gene PTEN, a lipid phosphatase responsible for dephosphorylating the 3′-phosphate group of PIP3 to generate PIP2 [13]. When PTEN is deficient, mutated, or inactivated, PI3K is activated, which promotes phosphorylation of AKT and drives cell proliferation and growth [14]. Our previous research has already revealed that exosomal miR-7 can reverse gefitinib resistance [15], and exosomal lncRNAs that regulate PTEN tumor suppressor activity in NSCLC cells harboring EGFR mutations remain poorly understood.

Exosomes are 30–150 nm extracellular vesicles, which are formed by intraluminal budding via the endosomal pathway [16]. Exosomes are produced and released by various cells, carry multiple bioactive substances, including nucleic acids, lipids, and proteins, and participate in communication between cells by acting as carrier protein-coding, for example, nucleic acids, from parent cells to target cells [17,18]. Long non-coding RNAs (lncRNAs) comprise a heterogeneous set of transcripts longer than 200 nucleotides, with minimal or no protein-coding potential [19]. Recent studies have validated that lncRNAs take part in multiple tumorigenic processes, for instance, cell proliferation, apoptosis, invasion, metabolism and drug resistance [20,21,22]. In the cytoplasm, lncRNA functions as competing endogenous RNA (ceRNA) and interacts with miRNA, resulting in the translation of transcripts targeted by miRNAs [23]. For example, Hsu et al. observed that exosome-transmitted lnc-MLETA1 facilitates lung cancer cell motility and invasion by sponging miR-497-5p and miR-186-5p [24]. Nevertheless, the role of exosomal lncRNAs in osimertinib resistance is not well known.

Following treatment with first-generation EGFR-TKIs, patients with *EGFR*-mutant NSCLC frequently acquire the T790M resistance mutation. Although subsequent administration of the third-generation TKI osimertinib achieves initial clinical benefit, disease progression ultimately recurs, which markedly compromises the survival outcomes of these patients. Thus, elucidating the resistance mechanisms underlying osimertinib failure is not only an urgent clinical challenge but also a critical imperative to improve the prognosis of patients with *EGFR*-mutant NSCLC. Herein, we addressed the roles of exosomal lncRNA H19 in osimertinib resistance, with a specific focus on its modulatory mechanism in the PI3K-PTEN-Akt pathway.

## 2 Materials and Methods

### 2.1 Cell Lines and Culture Methods

Human lung adenocarcinoma H1975 cells (EGFR L858R/T790M) were obtained from the American Type Culture Collection (ATCC; CRL-5908™, Manassas, VA, USA). Osimertinib-resistant H1975 cells (named as H1975R, CTCC-0281-NY) were built by Meisen CTCC (Zhejiang, China) through continuous culture in osimertinib (MCE, HY-1577, Monmouth Junction, NJ, USA) at concentrations of 2, 4, 6, 8, 10, 12.5, 15, 17.5, 20, 22.5, 25, 27.5, and 30 μM. H1975R cells maintain resistance to osimertinib through continuous low-dose exposure (10 μM). Osimertinib was resuspended in dimethyl sulfoxide (DMSO; Sigma-Aldrich, D4540, St. Louis, MO, USA). Cell lines were kept in Dulbecco’s modified Eagle medium (DMEM; Servicebio, G4511, Wuhan, China), enriched with 10% fetal bovine serum (FBS; Servicebio, G8003, Wuhan, China), and 1% penicillin-streptomycin (Servicebio, G4003, Wuhan, China). Cells were grown in a humidified environment at 37°C supplemented with 5% CO_2_. Cell lines employed in the study were subjected to STR authentication and verified to be mycoplasma-free.

### 2.2 Exosome Isolation

When H1975 and H1975R cells ranged 80% confluence (5 × 10^6^ cells per dish) in complete medium, they were washed thrice with 1 × PBS and kept in FBS depleted of exosomes (SBI, EXO-FBS-50A-1, Palo Alto, CA, USA) for 24 h. The conditioned medium was spun at 2000× *g* for 30 min at 4°C to separate cells and debris. The cell-free supernatant was harvested, and 0.5 volumes of total Exosome Isolation Reagent (Invitrogen™, Thermo Fisher Scientific, 4478359, Waltham, MA, USA) were added, and then culturing overnight at 4°C. Exosomes were harvested at 4°C via spun at 10,000× *g* for 1 h and dispersed in 1 × PBS.

### 2.3 Characterization of Exosomes

Exosome morphology was detected via transmission electron microscopy (TEM; JEOL, JEM1230, Tokyo, Japan). Briefly, exosomes were fixed in 2% osmium tetroxide for 2 h at 4°C and dehydrated for 20 min with rising concentrations of ethanol (50%, 70%, 80%, 90%, and 100%), infiltrated at a volume of 2:1 in acetone and embedding medium, and embedded. The sections were dyed using 2% uranyl acetate dihydrate for 15 min and lead acetate for 5 min. The dimension of exosomes was estimated via nanoparticle tracking analysis (NTA, ParticleMetrix, ZetaView, Germany). A 20 μL exosome sample was diluted 50-fold with 980 μL 1 × PBS. The diluted solution was loaded using a 1 mL syringe. Particle imaging was performed at a conductivity of 15,000 μS/cm sensed using ZetaView 8.04.02 software and ZetaView PMX 110 (Particle Metrix, Meerbusch, Germany).

### 2.4 Quantitative Reverse Transcription Polymerase Chain Reaction (qRT-PCR)

Total RNA was extracted from H1975 and H1975R cells and their exosomes, and mice subcutaneous xenografted tumor tissues using TRIzol reagent (Ambion, Life Technologies, 15596026, Austin, TX, USA), and supplemented with 200 μL of chloroform (Servicebio, G3014, Wuhan, China) to the lysate. The mixtures were reacted for 3 min at room temperature (RT), followed by spun at 12,000× *g* for 20 min at 4°C. Then, the upper supernatant (aqueous phase) was incorporated into 500 μL of isopropanol (Sangon Biotech, A600918, Shanghai, China), reacted at RT for 10 min, spun at 12,000× *g* for 20 min at 4°C, subsequently RNA was precipitated. Finally, RNA pellets were washed three times in 1 mL absolute ethyl alcohol and dissolved in RNase-free ddH_2_O. The RNA concentration and purity were measured via NanoDrop 2000 (Thermo Fisher Scientific, Waltham, MA, USA). A260/A280 ranging from 1.8–2.1 indicated high RNA purity.

1 μg of total RNA was blended with 5× All-in-one RT Buffer (4 μL) and All-in-one No RT Control Mix (1 μL), cDNA synthesis was performed at 50°C for 15 min followed by at 85°C for 5 s using the Toloscript all-in-one RT Easy Mix for qPCR Kit (TOLOBIO, 22107, Shanghai, China). 2 μL of cDNA was combined with 10 μL of 2 × Q3 SYBR qPCR Master Mix, 10 μM of forward primer and 10 μM of reverse primer. qPCR amplification was undertaken following 2 × Q3 SYBR qPCR master mix Kit instructions (TOLOBIO, 22204, Shanghai, China): pre-denaturation at 95°C for 30 s was applied, prior to 40 amplification cycles of 95°C for 10 s and 60°C for 30 s.

2 μg total RNA was combined with 10 μL of 2× miRNA RT Reaction Buffer and 2 μL of miRNA RT Enzyme Mix, then incubated at 42°C for 60 min to conduct miRNA poly(A) tailing and reverse transcription. Cultivation at 95°C for 3 min was employed to quench the reaction via enzyme deactivation, affording miRNA cDNA, following the miRcute Plus miRNA First-strand cDNA synthesis Kit (Tiangen, KR211, Beijing, China). 2 μL cDNA was combined with 10 μL 2 × miRcute Plus miRNA PreMix (SYBR&ROX), 10 μM forward primer and 10 μM reverse primer. cDNA amplification was performed with an initial denaturation at 95°C for 15 min, then 40 cycles at 94°C for 20 s and 60°C for 34 s, following the miRcute Plus miRNA qPCR Kit (SYBR Green) (Tiangen, FP411, Beijing, China).

Total RNA was reverse-transcribed to lncRNA cDNA via lnRcute lncRNA First-Strand cDNA Kit (Tiangen, KR202, Beijing, China). 2 μg total RNA was blended with 2 μL of 5 × gDNA Buffer, and cultured at 42°C for 3 min to eliminate genomic DNA. Next, the mixtures were supplemented with 10 × lnR RT Buffer (10 μL), lnR RT Enzyme Mix (1 μL) and lnR-RT Primer Mix (2 μL), and then cultivation at 42°C for 15 min and 95°C for 3 min, resulting in the production of lncRNA cDNA. 100 ng of cDNA was mixed with 10 μL of 2 × lnR lncRNA PreMix, 10 μM forward primer, 10 μM reverse primer and 1 μL 50× ROX Reference Dye. Amplification of cDNA was conducted employing the lnRcute lncRNA qPCR Kit (SYBR Green) (Tiangen, FP402, Beijing, China). Thermal cycling was initiated by pre-denaturation at 95°C for 15 min, with subsequent 40 cycles at 94°C for 20 s and 60°C for 34 s.

GAPDH and U6 were used for normalization. RNA primers were generated by Sangon Biotech. qRT-PCR was conducted via the Applied Biosystems 7500 sequence detection system (Thermo Fisher Scientific, Waltham, MA, USA). qRT-PCR reactions were conducted three times independently, and relative RNA expression was quantified with the 2^−^^ΔΔCt^ algorithm. The primers used are shown in Table 1.

**Table 1:** qPCR primer sequence.

Genes	Forward Primer (5′–3′)	Reverse Primer (5′–3′)
PI3K	agtcttccatgaaaacgtcac	gaatccaccgcccagatgtcaag
AKT	aacaacttct ctgtggcgcag	gctcctcaggagtctccacatg
PTEN	ggattcgacttagacttgacc	cggtgtcataatgtctttcagc
GAPDH	gatgacatcaagaaggtggtga	gttgtcataccaggaaatgagc
GAS5	agcacttgagcagctttcttctg	agcgcctggctcccccgcccgac
H19	caaagcctccacgactctgt	actcacgcacactcgtactg
MEG3	ctgaatcaccaaaggcacgc	gttgctctcatcgaccccat
NBATI	ttggggaggaagaccacaga	actaccacagccaggcaatc
U6	ctcgcttcggcagcaca	aacgcttcacgaatttgcgt
hsa-miR-338-3p	cgcgtccagcatcagtgatt	
hsa-miR-22-3p	cgaagctgccagttgaag	
has-miR-19b-3p	cgtgtgcaaatccatgc	
hsa-miR-21-5p	cgcgtagcttatcagactga	
hsa-miR-17-5p	cgcaaagtgcttacagtg	
hsa-miR-25-3p	cgcgcauugcacuugucucg	
hsa-miR-148-3p	cgcgucagugcacuacagaa	
hsa-miR-181-5p	cgcgaacauucaacgcuguc	

### 2.5 Western Blot

Total protein of cells, exosomes, and mice xenografted tumor tissues was solubilized by RIPA buffer (Beyotime, P0013B, Shanghai, China) added into phosphatase and proteinase inhibitors (Beyotime, P1045, Shanghai, China) for 40 min at 4°C. Then, lysate was spun at 12,000× *g* for 20 min at 4°C. The total protein was examined via BCA protein assay Kit (Keygen Biotech, KGP902, Nanjing, China). Protein samples (30 μg per lane) were **fractionated** via 10% SDS-PAGE and blotted onto PVDF membranes. Block using 5% skimmed milk at RT for 2 h, and then the membranes were cultured in primary antibodies overnight at 4°C, washed three times with PBST, probed with HRP-labeled anti-rabbit IgG (1:1000, Beyotime, A0208, Shanghai, China) at RT for 2 h. Protein signals were measured using an ECL substrate (Bio-Rad, 1705060, Shanghai, China), and protein intensity was analyzed via a ChemiDocTMMP imaging system (Bio-Rad, 1708280). GAPDH (1:5000, Affinity, AF7021, Jiangsu, China) served as the internal control. The anti-bodies used were rabbit anti-PTEN (1:2000, HUABIO, ET1606-43, Hangzhou, China), anti-p-ATK (1:1000, Affinity, AF00016, Jiangsu, China), anti-AKT (1:5000, HUABIO, ET1609-51), anti-CD9 (1:2000, HUABIO, HA721533), and anti-TSG101 (1:2000, HUABIO, ET1611-87). All western blot experiment was conducted in triplicate, and band intensities were monitored via ImageJ 1.8.0 (National Institutes of Health, Bethesda, MD, USA).

### 2.6 Cell Transfection

The cy5-siRNA (F, 5′-GGAGAGUUAGCAAAGGUGATT-3′, R, 5′-UCACCUUUGCUAAC UCUCCTT-3′), pcDNA3.1-H19 plasmid (F, FCMV-F 5′-CGCAAATGGGCGGTAGGCGTG-3′, R, hGH-PolyA 5′-ACTGGAGTGGCAACTTCCAGGGC-3′), and the corresponding negative controls (F, 5′-UUCUCCGAACGUGUCACGUTT-3′, R, 5′-ACGUGACACGUUCGGAGAATT-3′) were prepared by OBiO (Shanghai, China). The above-mentioned siRNA, pcDNA3.1-H19 plasmid were transfected via the Lipofectamine™ 2000 (Thermo Fisher Scientific, 11668019, Waltham, MA, USA) at 2.5 μg per well in 6-well plates. miR-148-3p mimics (F, 5′-UCAGUG CACUACAGAACUUUGU-3′, R, 5′-AAAGUUCUGUAGUGCACUGAUU-3′), NC mimics (F, 5′-UUCUCCGAACGUGUCACGUTT-3′, R, 5′-ACGUGACACGUUCGGAGAATT-3′), miR-148-3p inhibitors (5′-ACAAAGUUCUGUAGUGCACUGA-3′), and NC inhibitors (5′-CAGUACUUUUGU GUAGUACAA-3′) were constructed via GenePharma Co., Ltd. (Shanghai, China). The final transfection concentration of mimics and inhibitors was 20 μM.

### 2.7 Immunofluorescence

H1975R cells were introduced with the pcDNA3.1-H19-mCherry plasmids (OBiO, Shanghai, China), after 24 h, cell membrane was stained with 2 μM PKH67 (Sigma-Aldrich, MINI67-1KT, USA) at RT for 5 min. After 24 h, H1975 cells were maintained in the culture medium from H1975R cells for 48 h. Then, 20 μL of H1975 cells (500 cells) suspension was seeded into 24-well plate and incubated for 24 h. Then, cells were fixed with 4% paraformaldehyde at RT for 20 min, and the nuclei were **dyed** with 4′,6-diamidino-2-phenylindole (DAPI; 1 μg/mL) (Beyotime, P0131, Shanghai, China) at RT for 15 min. Cells were detected and taken pictures by LSM 900 laser scanning confocal microscope (Zeiss, Germany).

### 2.8 Dual-Luciferase Reporter Assay

The interaction sites of miR-148-3p on the lncRNA H19 3′UTR and PTEN 3′UTR were predicted via TargetScan (https://targetscan.org/vert_80) and lncBase (https://diana.e-ce.uth.gr/lncbasev3). First, we constructed the WT-H19 (PmirGLO-H19-WT, F: 5′-CTAGTTGTTTAAACGAGCTCGAGCTCCTCCAGCGGGATGA-3′, R: 5′-AGGTCGACTCTAGACTCGAGCAGCCGGCGCCCAGTCAC-3′) vector, Mut-H19 (PmirGLO-H19-Mut, F: 5′-CTGTTTCAGCAAAACGTGACCTTGGAGTTGTGGAGACG-3′, R: 5′-GGTCACGTTTTGCTGAAACAGCAAATTAAATTCAGAAGGG-3′) vector, WT-PTEN (PmirGLO-PTEN-WT, F: 5′-CTAGTTGTTTAAACGAGCTCACCTTGTCTTTCATAAAAGCTGAAAATTGTTA-3′, R: 5′-AGGTCGACTCTAGACTCGAGATTTCCAATGACTACACCATAAAATGTAAGC-3′) vector, and Mut-PTEN (PmirGLO-PTEN-Mut, F: 5′-TATTTCGGGATAACGTGACATATTATTTTTCCTTTGGAATGTGAAG-3′, R: 5′-AATAATATGTCACGTTATCCCGAAATAAATGTCATTATTTATGACCTGGC-3′) vector. Next, 0.15 μg of reporter vector and miR-148-3p (10 μM) were introduced into 293T cells (2 × 10^4^ cells/well) using 0.45 μL Hieff TransTM (Yeasen Biotech, 40802ES, Shanghai, China). Transfection for 6 h, subsequently, luciferase reporter activity was monitored with a dual-luciferase reporter assay system (Promega, E1910, Madison, WI, USA) in accordance with the product’s protocols. Finally, luciferase reporter activity was estimated via the firefly/Renilla luciferase signal in cells.

### 2.9 RNA Immunoprecipitation (RIP)

RIP assay was executed via BersinBio™ RNA immunoprecipitation (RIP) Kit (BersinBio, Bes5101, Guangzhou, China) in accordance with the product’s protocols. H1975R cells (1 × 10^7^) were gathered and dissolved in 100 μL of RIP lysis buffer supplemented with RNase inhibitor. IP lysis buffer was added to antibodies against 5 μg Ago2 (HUABIO, JF0992), with Mouse IgG (Proteintech, B900620, Wuhan, China) as a negative control. Then, lncRNA H19 and miR-148-3p were extracted from the immunoprecipitation complexes and subjected to reverse transcription and qPCR. Target RNA expression was normalized to the input control, and fold enrichment was evaluated via the 2^−^^ΔΔCt^ method with IgG control group values set as 1. Results are shown as mean ± standard deviation (SD) from three independent replicates.

### 2.10 CCK-8 Assay

H1975 and H1975R cells were administered into 96-well plate (all 100 μL/well, 8000 cells/well). After 24 h, 100 μL of osimertinib at gradient concentrations (0, 0.1, 1.0, 2.5, 5.0, 10, 20 μM) was added, and then incubation for another 24 h. Each concentration was set up in triplicate. Cells were incubated with 10 μL CCK8 kit (NCM Biotech, C6050, Jiangsu) for 1 h. Absorbance was calculated at 450 nm via the SpectraMax iD5 microplate spectrophotometer (Molecular Devices, San Jose, CA, USA). Cell viability (%) = (OD_treat_ − OD_blank_)/(OD_untreated control_ − OD_blank_) × 100%.

### 2.11 Colony Formation Assay

H1975 and H1975R cells were cultured in 12-well plates (1000 cells/well) to observe colony formation. After 24 h, osimertinib at various concentrations was added, with three repeated wells for per drug. The cells were maintained for 7–14 days until visible colony formation, at which point the culture was terminated. Then, the cells were fixed with 4% paraformaldehyde (Servicebio, G1101) for 20 min at RT, and dyed via 0.1% crystal violet (Sigma, V5265) for 15 min at RT. Colonies containing ≥ 50 cells were considered positive colonies.

### 2.12 Cell Apoptosis

Apoptosis of H1975 and H1975R cells was examined via annexin V-FITC apoptosis detection Kit (Keygen Biotech, KGA107). Cells were digested with pancreatic enzymes free of EDTA, spun at 2000× *g* for 4 min, and gathered 1 × 10^5^ of cells. After supplementation with 500 μL of Binding Buffer (Keygen Biotech, KGA107) and 5 μL of annexin V-FITC, the mixture was maintained in darkness for 5 min at RT. Apoptosis percentage was determined by BD FACSCanto™ II flow cytometry, which relies on a 488 nm blue laser, and fluorescence parameters are FITC-A and PI-A, the light scatter parameters are FSC-A and SSC-A. Data were monitored by BD FACSDiva™ software v9.1 (BD Biosciences, San Jose, CA, USA).

### 2.13 TUNEL Apoptosis Detection (Colorimetric)

Frozen sections (5 μm thickness) of mice tumor tissue were baked for 20 min in oven at 37°C, then fixed with methanol for 30 min, shaken, and washed three times on a decolorizing shaker in PBS. 20 μg/mL of proteinase K digested proteins on the sections at 37°C for 20 min, allowing TdT enzyme to enter nucleus and label fragmented DNA. Then, sections were maintained in 3% H_2_O_2_ for 20 min at RT to quench endogenous peroxidase. Tumor tissue apoptosis was assessed via DAB (SA-HRP) TUNEL cell apoptosis detection Kit (Servicebio, G150). Images were captured by a BX53 microscope (Olympus, Tokyo, Japan).

### 2.14 Ki67 Cell Proliferation Assay (IHC)

Frozen sections (5 μm thickness) of mouse tumor tissue were dried at RT, baked for 20 min in oven at 37°C, fixed in methanol for 20 min, shaken, then washed three times on a decolorizing shaker in 1 × PBS. Next, antigen retrieval was carried out and sections were fixed with 3% H_2_O_2_ for 10 min to neutralize endogenous peroxidase and blocked by normal goat serum (Bio-Rad, C07SA, Shanghai, China) at RT for 30 min. The primary antibody used was rabbit anti-Ki-67 (1:50, Servicebio, GB111141) overnight at 4°C. Goat anti-rabbit IgG (1:300, Servicebio, GB21303) was employed as the secondary antibody, incubated at RT for 30 min. Images were detected via BX53 microscope (Olympus, Tokyo). The proportion of Ki-67-expressing cells was counted in at least 500 tumor cells.

### 2.15 Xenograft Animal Model

Eighteen four-week-old, 20 g, male Balb/C nude mice were obtained from Slack Laboratory Animal Co. (Shanghai, China). 200 μL PBS containing 1 × 10^7^ H1975R cells was subcutaneously implanted into the dorsal flanks of the mice. On day 2, mice were marked with ear tags. The body mass of mice was detected every other day. The tumor size was detected by vernier caliper every other day. After 20 days, mice were randomly assigned to three groups (*n* = 6), Group1: H1975R/Mock; Group2: H1975R + osimertinib (5 mg/kg), mice were intragastrically treat with osimertinib (5 mg/kg) daily; Group3: H1975R + exosomes-H1975 (3 μg) + osimertinib (5 mg/kg), mice were intratumorally injected with exosomes derived from H1975 cells (3 μg) three times a week, and intragastrically treat with osimertinib (5 mg/kg) daily. Tumor volume (mm^3^) = length × width^2^ × 1/2. Mice were sacrificed by carbon dioxide inhalation asphyxiation when the diameter of a single tumor exceeded 15 mm, and the xenografted tumors were subjected to histological analysis. No mice were excluded from the experiment.

Animal experiments were undertaken pursuant to the National Institutes of Health (NIH) Guide for the Care and Use of Laboratory Animals. The animal experiments were authorized by the Institutional Animal Ethics Committee of Yueyang Hospital of Traditional Chinese and Western Medicine affiliated with Shanghai University of Traditional Chinese Medicine (Approval No YYLAC-2024-270-1).

### 2.16 Statistical Analysis

The experimental data were statistically evaluated by GraphPad Prism version 10 (GraphPad Software Inc., Boston, MA, USA). Significant differences between two groups were measured by Student’s *t*-test, and comparisons between multiple groups (>2) were conducted by one-way analysis of variance (ANOVA). Experiments were performed in triplicate. Results are shown as mean ± SD, and *p* < 0.05 was regarded as statistically meaningful.

## 3 Results

### 3.1 lncRNA H19 Is Related to Osimertinib Resistance

**Figure 1:**
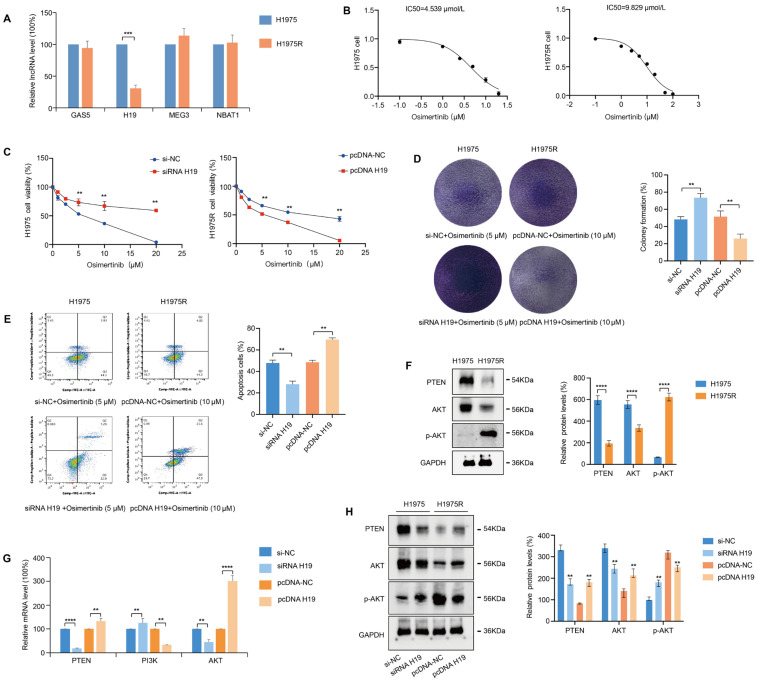
lncRNA H19 as a suppressor in osimertinib resistance. (**A**) Quantitative reverse transcription polymerase chain reaction (qRT-PCR) measured significant differences in expression of lncRNA between H1975 and H1975R cells. (**B**) CCK-8 assay estimated the inhibitory concentration 50 (IC50) of osimertinib for H1975 and H1975R cells following 24 h treatment. (**C**) H1975 cells were transfected using siRNA-H19 or si-NC, H1975R cells were transfected using pcDNA-H19 or pcDNA-NC plasmids. CCK-8 assay estimated the cell viability via osimertinib treatment with increasing concentrations. (**D**) Cell clonogenic growth was observed via colony formation assay. (**E**) Cell apoptosis was monitored via annexin V/PI assay. (**F**) Western blotting detected the expression of phosphatase and tensin homolog (PTEN), protein kinase B (AKT), and p-AKT proteins in H1975 and H1975R cells. (**G**) qRT-PCR confirmed that knockdown or overexpression of lncRNA H19 modulated the PI3K-PTEN-AKT signaling pathway. (**H**) Western blotting examined the roles of lncRNA H19 overexpression or knockdown on protein expression of PTEN, AKT and p-AKT. **p < 0.01, ***p < 0.001, ****p < 0.0001. Values are shown as mean ± standard deviation (SD).

In an attempt to explore lncRNAs that participate in osimertinib resistance, we read numerous studies and screened out the lncRNAs that regulate PTEN expression in cancer cells, including GAS5 [25,26], H19 [27,28], MEG3 [21], and NBAT1 [29]. In contrast to the H1975 cells, the lncRNA H19 expression level was decreased dramatically in H1975R cells, and GAS5, MEG3, and NBAT1 exhibited no significant difference (Fig. 1A). First, to research the function of lncRNA H19 in osimertinib resistance, H1975R and H1975 cells were administered gradually increasing concentrations of osimertinib, CCK-8 assay examined the half maximal inhibitory concentration (IC50), the IC50 of H1975R cells was 2-fold higher than H1975 cells (Fig. 1B). Next, we overexpressed lncRNA H19 in H1975R cells by transfection with pcDNA-H19 plasmids, H1975 cells were transfected with an siRNA to knockdown H19. CCK-8 assay revealed that H1975 cell proliferative activity enhanced after transfection using siRNA H19, but overexpression of H19 reduced the proliferation of H1975R cells with osimertinib treatment (Fig. 1C). Correspondingly, the colony formation (Fig. 1D) and annexin V/PI apoptosis assays (Fig. 1E) showed that pcDNA H19 reduced colony formation of H1975R cells and enhanced cell apoptosis, whereas siRNA H19 increased colony formation of H1975 cells and suppressed cell apoptosis.

PI3K-Akt is the major downstream signaling of EGFR, when PTEN is lost, PI3K is activated, leading to acquired resistance to osimertinib [14]. Accumulating evidence indicates that lncRNA H19 modulates PI3K-Akt signaling pathway activation in multiple diseases [30]. Western blotting displayed that expression of PTEN was remarkably decreased in H1975R cells, and PI3K-Akt signaling was activated in H1975R cells (Fig. 1F). qRT-PCR showed that siRNA H19 increased PI3K expression and reduced PTEN and AKT expression in H1975 cells (Fig. 1G). Further, western blotting displayed that phosphorylation level of AKT was reduced after transfection with pcDNA H19 plasmid in H1975R cells, but was augmented after transfection with siRNA in H1975 cells (Fig. 1H). These results suggest that lncRNA H19 affects osimertinib resistance through the PI3K-AKT pathway.

### 3.2 LncRNA H19 Functions as a ceRNA Sponging miR-148-3p to Regulate PTEN Levels

**Figure 2:**
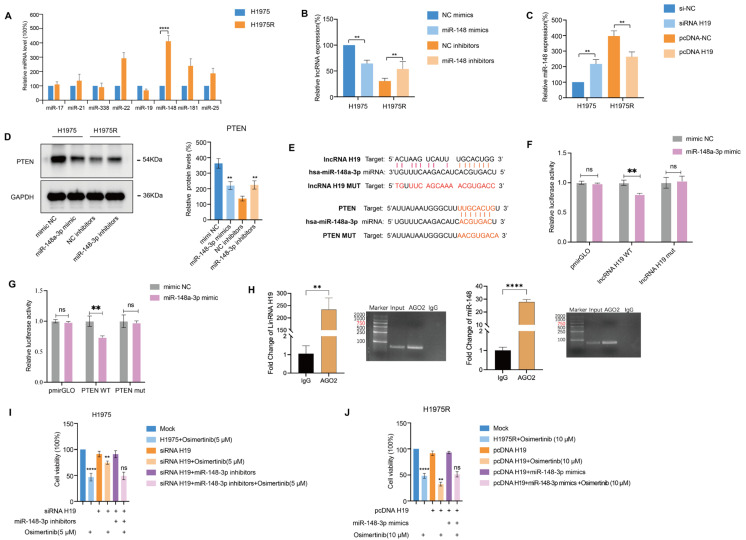
PTEN was proven as a target of miR-148-3p and is modulated via lncRNA H19. (**A**) qRT-PCR **uncovered** that level of miR-148-3p was upregulated in H1975R cells relative to H1975 cells. (**B**) lncRNA H19 levels was detected by qRT-PCR following miR-148-3p knockdown or overexpression in lung cancer cells. (**C**) The expression level of miR-148-3p was measured by qRT-PCR following H19 knockdown or overexpression lung cancer cells. (**D**) Western blotting examined the actions of miR-148-3p knockdown or overexpression on PTEN transcription. (**E**) Schematic of predicted miR-148-3p target sequences in the lncRNA H19 3′ UTR and PTEN 3′ UTR. (**F**) Luciferase reporter system proved that miR-148-3p binds to lncRNA H19. (**G**) Luciferase reporter system proved that miR-148-3p binds to PTEN. (**H**) RNA immunoprecipitation (RIP) experiment was conducted in H1975R cells and coprecipitated RNA functioned to quantify H19 and miR-148 expression. (**I**) Following 24 h transfection with siRNA H19, H1975 cells were further transfected by miR-148-3p inhibitors and exposed to 5 μM osimertinib for 24 h. CCK8 assay observed the proliferative activity of H1975 cells. (**J**) H1975R cells were first transfected using pcDNA-H19 plasmid, after 24 h, then transfected using miR-148-3p mimics, and incubated with 10 μM osimertinib for 24 h. CCK-8 assay observed the proliferative activity of H1975R cells. ns: no significance. **p < 0.01, ****p < 0.0001. Values are shown as mean ± SD.

lncRNA H19 has been shown to suppress airway inflammation through upregulation of PTEN expression via the ceRNA mechanism [27]. We assumed that certain miRNAs could bind to the targeted transcripts, inhibiting the translation of mRNA into the PTEN protein in H1975R cells. To verify this assumption, we analyzed bioinformatics database containing TargetScan (https://targetscan.org/vert_80), lncBase (https://diana.e-ce.uth.gr/lncbasev3) and miRbase (https://www.mirbase.org/), and screened several miRNAs (miR-17-5p, miR-338-3p, miR-21-5p, miR-22-3p, miR-19b-3p, miR-148-3p, miR-181-5p, and miR-25-3p) that might be related to osimertinib resistance. Among these miRNAs, miR-148-3p had the highest expression in H1975R cells (Fig. 2A). Subsequently, the miR-148-3p mimics were transiently transfected to H1975 cells, and lncRNA H19 expression was obviously attenuated compared to the NC mimics group. Additionally, the miR-148-3p inhibitor was transiently transfected into H1975R cells, and lncRNA H19 expression was remarkably enhanced in contrast to the NC inhibitor group (Fig. 2B). Moreover, transfection of pcDNA H19 in H1975R cells, miR-148-3p expression was suppressed, which was enhanced after transfection with siRNA H19 in H1975 cells (Fig. 2C). Western blotting observed that after H1975 cells were transfected using miR-148-3p mimics, PTEN transcription was reduced, whereas after H1975R cells were transfected using miR-148-3p inhibitors, the PTEN protein was increased (Fig. 2D). Thus, miR-148-3p negatively regulated PTEN protein expression.

We forecasted the interaction sites of miR-148-3p on the lncRNA H19 3′UTR and PTEN 3′UTR using TargetScan and lncBase (Fig. 2E). This prediction was verified by dual-luciferase reporter system. The luciferase reporter activity of H19-WT in the miR-148-3p mimic was remarkably decreased relative to the NC-mimic, but no statistically significant distinction was detected in the H19-Mut group, **revealing** that H19 was a target of miR-148-3p (Fig. 2F). The luciferase reporter activity of PTEN-WT was obviously decreased via the miR-148-3p mimic relative to that of the NC-mimic, while no significant distinction was detected in the PTEN-Mut group, suggesting that PTEN was a target gene of miR-148-3p (Fig. 2G). It has been proven that gene silencing of miRNAs occurs by recruiting nuclear-localized Ago2. The presence of lncRNA H19 and miR-148-3p in Ago2 immunoprecipitants was detected using RNA immunoprecipitation (RIP). In contrast to the IgG control group, both H19 and miR-148-3p were gathered in Ago2 pellets, suggesting that lncRNA H19 could interact with miR-148-3p by binding to Ago2 (Fig. 2H).

In addition, transfection of H1975 cells using siRNA H19, **subsequently**, cells were transfected using miR-148-3p inhibitors and exposed to 5 μM osimertinib. CCK8 assay observed the cell viability of H1975 cells. As show in Fig. 2I, silencing H19 in H1975 cells reduced sensitivity to osimertinib and enhanced cell proliferation. Co-transfection with miR-148-3p inhibitors decreased cell proliferation, thus counteracting the proliferative effect of siRNA H19. H1975R cells were transfected with the pcDNA H19 plasmid, followed by transfection using miR-148-3p mimics and treatment with 10 μM osimertinib. Overexpression of H19 in H1975R cells enhanced the osimertinib cytotoxicity. Transfection of miR-148-3p mimics into H1975R cells enhanced cellular proliferation and facilitated resistance to osimertinib (Fig. 2J). Overexpression of H19 suppressed cell proliferation, while overexpression of miR-148-3p exerted the opposite effect, indicating that H19 may bind to miR-148-3p and counteract each other’s function. Taken together, our results revealed that lncRNA H19 functions as a molecular sponge targeting miR-148-3p, by this means modulating PTEN translation.

### 3.3 Characterization of Exosome

**Figure 3:**
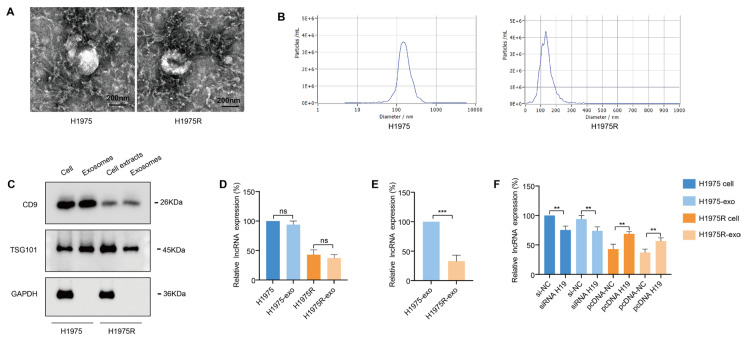
Extracellular lncRNA H19 was incorporated into exosome. (**A**) Transmission electron microscopy (TEM) images showed the morphological features of exosomes released by H1975 and H1975R cells. (**B**) Nanoparticle tracking analysis (NTA) measured the **dimension** of exosomes. (**C**) Western blotting detected the protein markers of exosomes CD9, TSG101. (**D**) qRT-PCR examined the lncRNA H19 expression levels in cells and exosomes. (**E**) qRT-PCR examined that the level of exosomal lncRNA H19 was downregulated in H1975R cells relative to H1975 cells. (**F**) qRT-PCR examined that lncRNA H19 expression in both exosomes and cells following transfection with pcDNA-H19/pcDNA-NC plasmids or siRNA-H19/si-NC. ns: no significance. **p < 0.01, ***p < 0.001. Values are shown as mean ± SD.

To dissect the potential functions of exosomes in osimertinib resistance, we purified exosomes from the conditioned media (CM) of H1975 and H1975R cells via ultracentrifugation. TEM revealed that exosomes were cup-shaped, round, or oval vesicles (Fig. 3A). The diameter of the exosomes from H1975 and H1975R cells ranging from 100 to 150 nm was detected by NTA (Fig. 3B). Western blotting indicated the presence of CD9 and TSG101 markers in the exosome samples, but GAPDH was not detected (Fig. 3C). These data suggested that the membrane vesicles isolated from H1975 and H1975R cells were exosomes. The lncRNA H19 in cell extracts and exosomes was quantitatively determined by qRT-PCR, which **uncovered** exosomal lncRNA H19 level was nearly equal to that in cell extracts (Fig. 3D), and expression of lncRNA H19 was significantly elevated in exosomes derived from H1975 cells relative to H1975R cells (Fig. 3E). In addition, we transfected H1975 cells with siRNA-H19 or si-NC, and H1975R cells with pcDNA-H19 or pcDNA-NC plasmids. H19 expression was obviously decrease in H1975 cells and their exosomes after transfected using siRNA, while the expression of H19 was enhanced in H1975R cells and their exosomes following transfected using pcDNA-H19 plasmids (Fig. 3F). These data indicate that exosomes are the main carriers of the extracellular lncRNA H19.

### 3.4 Exosome Transferred lncRNA H19 Regulated Osimertinib Resistance through miR-148-3p/PTEN Axis

**Figure 4:**
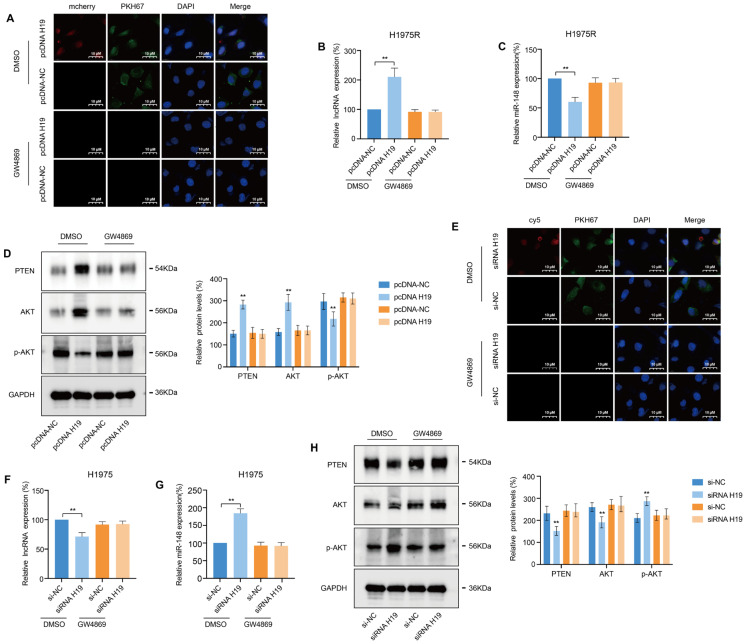
The transfer of lncRNA H19-containing exosomes between H1975 and H1975R cells. (**A**) H1975 cells transfected with mcherry-pcDNA-H19 or mcherry-pcDNA-NC plasmid, and cell membranes stain with PKH67 dye. Culture media (CM) from H1975 cells pretreated with DMSO or GW4869 was collected and added to H1975R cells for 48 h. Fluorescent signals in H1975R cells were monitored by confocal microscopy. (**B**) lncRNA H19 expression in H1975R cells was measured via qRT-PCR. (**C**) miR-148-3p expression in H1975R cells was monitored via qRT-PCR. (**D**) Western blot served to monitor the proteins levels in the PTEN-AKT signaling in H1975R cells. (**E**) H1975R cells transfected with cy5-siRNA-H19, and cell membranes stain with PKH67 dye. Conditioned medium from H1975 cells pretreated with DMSO or GW4869 was collected and supplemented with H1975 cells for 48 h. Fluorescent signals were observed under confocal microscopy. (**F**) qRT-PCR quantified lncRNA H19 expression levels in H1975 cells. (**G**) qRT-PCR quantified miR-148-3p expression levels in H1975 cells. (**H**) Western blotting measured the protein expression in PTEN-AKT signaling in H1975 cells. **p < 0.01, Values are shown as mean ± SD.

To confirm whether lncRNA H19 can be delivered between cells by exosomes, H1975 cell was transfected by mcherry-pcDNA-H19 or mcherry-pcDNA-NC plasmids and cytomembrane labelled with PKH67 dye. After 24 h, H1975R cells were incubated with CM from the H1975 cells for 48 h. Confocal microscopy revealed a red fluorescence signal in the pcDNA H19 group but not in the pcDNA-NC group, while green fluorescence signals were detected in both groups (Fig. 4A). To further identify the roles of exosome, the production of exosomes was prevented by GW4869 on neutral sphingomyelinase-2 (nSMase). After H1975 cells were treated with GW4869, there are no fluorescence signal was detected in H1975R cells (Fig. 4A). Additionally, qRT-PCR determined that lncRNA H19 levels were markedly increased in H1975R cells after H1975 cells were incubated with DMSO. After blocking the secretion of exosomes, lncRNA H19 expression did not differ significantly (Fig. 4B), indicating that H19 were transmitted from H1975 cells to H1975R cells via PKH67 labelled-exosomes. Subsequently, miR-148-3p level was detected, and it was significantly downregulated in H1975R cells incubated with H1975-CM containing pcDNA-H19 plasmids (Fig. 4C). When H1975R cells were treated with H1975-CM, western blotting **confirmed** that overexpression of H19 enhanced PTEN proteins expression in H1975R cells, whereas p-AKT levels decreased and AKT levels increased (Fig. 4D). These data suggest that exosomes transmitted lncRNA H19 from H1975 cells to H1975R cells can competitively bind to miR-148-3p to augment the translation of PTEN in H1975R cells, inhibiting the phosphorylation of AKT, thereby reversing osimertinib resistance.

To explore whether exosomal lncRNA H19 can be transferred to H1975 cells, H1975 cells were incubated in conditioned medium from H1975R cells transiently transfected with cy5-siRNA-H19 and labelled with PKH67 dye. Red and green fluorescence signals were detected in H1975 cells (Fig. 4E), suggesting that the siRNA-contained exosomes can be absorb by H1975 cells. Furthermore, we extracted RNA from H1975 cells, and qRT-PCR **examined** that lncRNA H19 expression level was markedly reduced and miR-148-3p expression increased in DMSO group, but not in GW4869 group (Fig. 4F,G). Nevertheless, the level of PTEN protein was decreased in DMSO group (Fig. 4H), indicating that siRNA of H19 inhibited translational activity of PTEN via sequestering miR-148-3p and activating the PI3K-Akt signaling in H1975 cells, leading to osimertinib resistance.

### 3.5 Exosomes Carrying lncRNA H19 Restrained the Resistance Phenotype of Osimertinib

**Figure 5:**
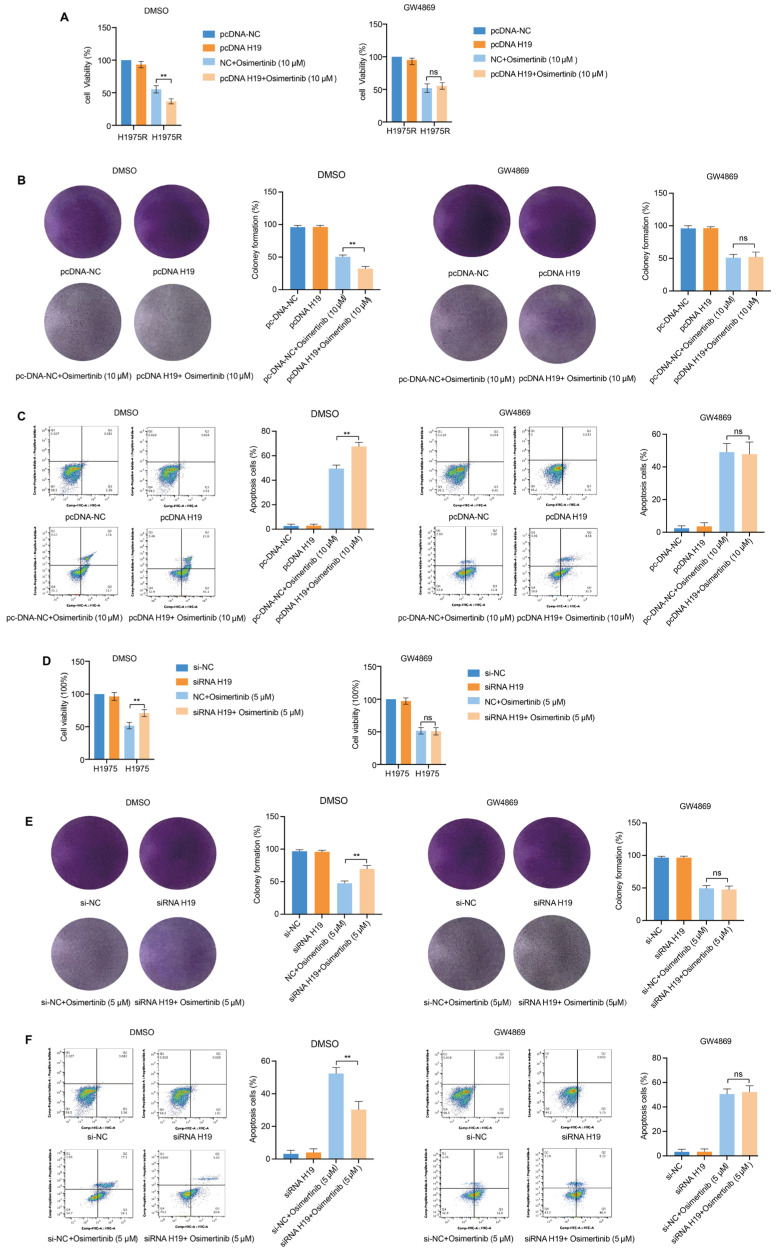
Exosomes transmitted lncRNA H19 restrained osimertinib resistance phenotype. (**A**) H1975R cells were co-incubated for 48 h with CM from H1975 cells transfected using pcDNA-H19 or pcDNA-NC plasmids and pretreated with DMSO or GW4869, CCK8 assay showed cell viability in H1975R cells exposed to 10 μM osimertinib for 24 h. (**B**) Colony formation assay evaluated cell clonal growth in H1975R cells. (**C**) Cell apoptosis was examined by the annexin V/PI assay in H1975R cells. (**D**) H1975 cells were co-cultured for 48 h in CM from H1975R cells transfected using siRNA-H19 or si-NC and pretreated with DMSO or GW4869. CCK-8 test measured cell proliferation viability after exposed to 5 μM osimertinib for 24 h. (**E**) Colony formation assay evaluated cell clonal growth in H1975 cells. (**F**) Cell apoptosis was examined via the annexin V/PI assay in H1975 cells. ns: no significance. **p < 0.01. Values are shown as mean ± SD.

To determine whether exosome-transferred lncRNA H19 could affect the osimertinib-resistant phenotype, H1975R cells were treat with the CM of H1975 cells transfected using pcDNA-H19 or pcDNA-NC plasmids and expose to 10 μM osimertinib. CCK-8 and clonogenic assays found that pcDNA-H19 markedly attenuated cell viability and colony growth in H1975R cells within the DMSO group, but the GW4869 group did not show any difference (Fig. 5A,B). The annexin V-FITC apoptosis assay showed that pcDNA H19 significantly enhanced apoptosis of H1975 cells in DMSO group, whereas not in GW4869 group (Fig. 5C). The above results suggested that exosomes transferred lncRNA H19 to H1975R cells that exhibited enhanced sensitivity to osimertinib treatment.

We further verified whether exosomes transferred lncRNA H19 from H1975R cells to H1975 cells can promote osimertinib resistant, H1975R cell transfected with siRNA or si-NC, then, H1975 cells were incubated in H1975R-CM and exposed to 5 μM osimertinib. CCK8 and clonogenic assays observed that exosomes carrying siRNA markedly promoted cell proliferation and clonal growth in H1975 cells (Fig. 5D,E). The annexin V/PI apoptosis assay revealed that siRNA-loaded exosomes significantly inhibited apoptosis in H1975 cells (Fig. 5F). Thus, exosomes mediated the transfer of siRNA-transfected lncRNA H19 from H1975R cells to H1975 cells, promoting the co-incubated of parental cells to obtain osimertinib resistance.

### 3.6 Exosomal lncRNA H19 Reversed Osimertinib Resistance In Vivo

**Figure 6:**
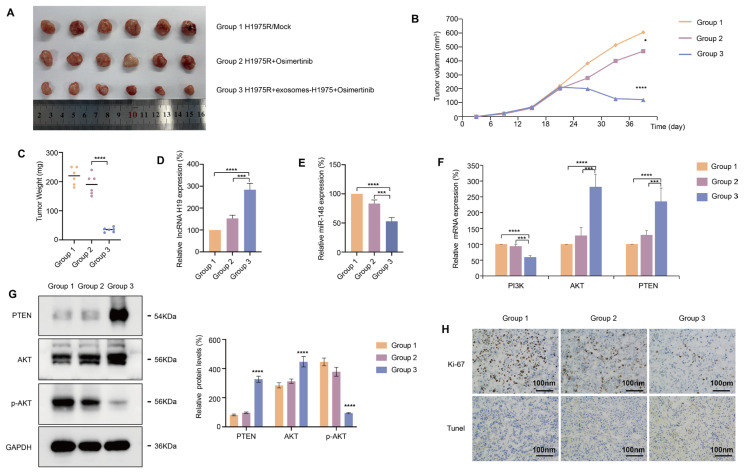
Exosomal lncRNA H19 reversed osimertinib resistance *in vivo*. (**A**) Tumor volume and mass of xenografts in nude mice subcutaneously inoculated with H1975R cells, with or without H1975 cell-derived exosomes (*n* = 6). (**B**) Curve of tumor volume changes with different treatments in nude mice (*n* = 6). (**C**) Tumor weight in three groups at the endpoint (*n* = 6). (**D**) lncRNA H19 expression in subcutaneous tumor tissues was evaluated via qRT-PCR. (**E**) miR-148-3p expression in subcutaneous tumors was examined via qRT-PCR. (**F**) qRT-PCR analysis of PI3K-PTEN-AKT signaling expression in xenograft tumors. (**G**) Western blotting examined the expression level of PTEN, AKT and p-AKT proteins in xenograft tumors across three groups. (**H**) Ki-67 (Immunohistochemistry, IHC) and Tunel assays showed tumor proliferation and promoted tumor apoptosis in xenograft tumors across three groups (Scale bar, 100 nm). *p < 0.05, ***p < 0.001, ****p < 0.0001. Values are shown as mean ± SD.

To elucidate the roles of exosomal lncRNA H19 in osimertinib resistance, we subcutaneously injected H1975R cells into the dorsal flanks of nude mice to form orthotopic xenograft models. On 20th days, according to different therapeutic interventions divide into groups, Group 1 was H1975R/Mock, and Group 2 mice were intragastrically administered osimertinib (5 mg/kg) daily. Group 3 was intratumorally injected into exosomes (3 μg) derived from H1975 cells twice a week, and treated with osimertinib (5 mg/kg) by intragastric administration every day. Xenograft tumor weight and volume in Group 3 were markedly small compared to Group 1 and Group 2, and Group 2 was slightly smaller than Group 1, with no statistical difference (Fig. 6A–C), suggesting that the injection of H1975 cell-derived exosomes into the xenograft tumors remarkably promoted osimertinib-mediated suppression of tumor weight and volume. qRT-PCR detected the lncRNA H19 expression level in xenograft tumor tissues, showed that lncRNA H19 level was markedly upregulated in Group 3 compared with Group 1 and Group 2, revealing that H1975 cell-derived exosomes increase lncRNA H19 expression (Fig. 6D). qRT-PCR measured that the miR-148-3p level in xenograft tumor tissues was markedly reduced in Group 3 instead of in Group 1 and Group 2 (Fig. 6E). Furthermore, we detected the activation of PTEN-Akt pathway in xenograft tumor tissues by qRT-PCR and western blotting. Osimertinib alone could not effectively upregulate PTEN and inhibit p-AKT, and combined treatment with H1975 cell-derived exosomes and osimertinib significantly increased PTEN expression and reduced p-AKT levels (Fig. 6F,G). Additionally, compared to Group 1 and Group 2, IHC analysis showed that significant improved positive areas of Ki-67 in xenografted tumor tissues in Group 3 (Fig. 6H). Tunel testing found that the positive areas of the TUNEL in xenograft tumor tissues were remarkably enhanced in Group 3 instead of in Group 1 and Group 2 (Fig. 6H). Ki-67 (IHC) and TUNEL assays demonstrated that osimertinib combined with H1975 cell-derived exosomes suppressed tumor proliferation and promoted tumor apoptosis. Altogether, we validated that exosomal lncRNA H19 reverses osimertinib resistance through competitive binding with miR-148-3p to upregulate PTEN *in vivo*.

## 4 Discussion

EGFR T790M confers resistance by blocking the competitive interaction of first- and second-generation TKIs within the EGFR ATP-interacting pocket [7,10]. However, osimertinib can irreversibly bind to EGFR kinase via a cysteine-797 residue targeting the ATP-interacting site by forming covalent bonds, thereby inhibiting EGFR-TKI-mediated downstream signaling [31]. Osimertinib can specifically interact with EGFR-activating mutations or the T790M resistance mutation, with impressive therapeutic efficacy in lung cancer patients [2,9,10,32]. However, all the patients eventually developed osimertinib resistance. Recent studies have that uncovered the C797S mutation and c-MET amplification are the major resistance mechanisms of osimertinib [33,34,35]. In order to extend the survival of *EGFR*-mutant lung cancer patients, a deeper understanding of the mechanisms driving acquired resistance is essential.

PTEN is almost prevalent inactivated tumor suppressor gene in malignant neoplasms, depends on lipid phosphatase activity, and exerts tumor suppressive effects, which negatively regulate the PI3K-Akt signaling [36]. A clinical study explored the correlation between deficiency of PTEN and therapeutic effects of EGFR-TKIs in patients with *EGFR*-mutant, found that the prognosis of ORR, PFS, and OS in patients with PTEN loss was worse [37]. Because PI3K-Akt signaling is the downstream of EGFR pathway, Yamamoto et al. strongly suggested that the absence of PTEN contributes to the resistance of NSCLC to gefitinib and erlotinib [38]. In addition, PTEN was activated at the G2/M phase to inhibit AKT/mTOR pathway in NSCLC, triggering growth arrest, senescence, autophagy, and apoptotic cell death [39]. In our study, the level of PTEN was remarkably reduced in osimertinib-resistant H1975R cells and the PI3K-Akt signaling was activated. Thus, PTEN loss is crucial for the development of osimertinib resistance.

Research suggests that lncRNAs are extensively expressed in malignant cells and contribute significantly to tumorigenesis [40]. In our study, the well-characterized lncRNA H19 was reduced in osimertinib-resistant H175R cells and suppressed the resistant phenotypes. The lncRNA H19 gene, on human chromosome 11p15.5, predominantly encodes cytoplasmic 2.3 kilobase long capped, spliced, and polyadenylated ncRNA, and exerts vital functions in regulating embryonic development and tumorigenesis [41,42]. Liu et al. revealed that lncRNA H19 facilitated tumor-related macrophages polarization towards the immunosuppressive M2 phenotype, resulting in pancreatic tumor cells growth and metastasis [43]. Xu et al. observed that lncRNA H19 was obviously reduced in lung cancer cells with resistance to EGFR-TKIs, and a high-level of H19 increased erlotinib cytotoxicity through induced ferroptosis [44]. Our researches showed that the overexpression of H19 restrained osimertinib resistance, whereas siRNA of H19 in H1975 cells enhanced the resistant phenotypes, indicating that H19 plays important roles in osimertinib resistance, and inhibit H1975R cells proliferation and promote apoptosis. lncRNA H19 is both an oncogenic and a tumor-suppressive gene, regulates multiple biologic functions, including the imprinted Igf2/H19 tandem locus, H19 antisence RNA, modular scaffold, encoding miR-675, and act as a ceRNA [45]. This study focused on elucidating how H19 functions as a ceRNA to modulate osimertinib resistance and influence the cellular drug-resistant phenotype.

Moreover, the knockdown of H19 reduced the PTEN expression and activated PI3K-Akt signaling in H1975 cells. In the cytoplasm, lncRNAs can complexes with mRNA, miRNA, and proteins to modulate post-transcriptional modifications [46]. In this study, we confirmed that lncRNA H19 is principally located in the cytoplasm and cooperates with Ago2 in osimertinib-resistant H1975R cells, indicating that H19 could act as an endogenous miRNA sponge. Combined bioinformatics prediction and luciferase reporter **analysis** demonstrated that miR-148-3p targets lncRNA H19. Overexpression of lncRNA H19 was accompanied by a significant downregulate in miR-148-3p expression in H1975R cells. We utilized bioinformatics to predict the targets of PTEN, and then confirmed by luciferase reporter **analysis**, which verified PTEN as the primary target of miR-148-3p and that miR-148-3p directly regulates PTEN transcription. Furthermore, miR-148-3p modification restored the effects of H19 suppression. Our research revealed that lncRNA H19 binds to miR-148-3p and modulates PTEN protein levels in osimertinib-resistant cells. Specifically, H19 exerts a suppressor function by sponging miR-148-3p in osimertinib-resistant cells. Our study revealed that lncRNA H19 functions as a tumor suppressor gene to restrain osimertinib resistance.

Furthermore, we investigated whether extracellular lncRNA H19 could inhibit osimertinib resistance by integrating it into exosomes. Exosomes as a novel means of information communication between cells and exert crucial functions in the pathogenesis of diseases and normal physiological processes [47]. Exosomes loaded with cytoplasmic and membranous matter originating from their parental cells. When exosomes are transferred to recipient cells from the host, the recipient cells can accurately capture the exosomes through the surface receptors of exosomes, resulting in the exchange of genetic information [48]. Exosomes are able to transfer bioactive substances to target cells efficiently and have been demonstrated to be an efficient transport tool to treat the disease [49]. Exosomal lncRNA is important regulator of cancer initiation and development, and implicated in multiple disease processes, such as malignant proliferation, angiogenesis, immune regulation, metastatic spread, and drug resistance [50]. In our study, exosomes carrying lncRNA H19 can be transferred between osimertinib-sensitive and-resistant cells and taken in by recipient cells, triggering the H19-based signal transduction in recipient cells. The miR-148-3p level in recipient cells was affected by exosomal lncRNA H19, and overexpression of H19 in recipient cells could sponge more miR-148-3p and lead to more PTEN protein being translated, inactivating of PI3K-Akt signaling in recipient cells. Our results suggest that exosomes carrying the lncRNA H19 from H1975 cells to H1975R cells restrain osimertinib resistance. Moreover, we also found that the lncRNA H19 loaded in exosomes could reverse the resistance phenotype in H1975R cells. *In vivo*, we discovered that the injection of exosomes from H1975 cells into xenograft tumors significantly enhanced osimertinib-mediated reduction in tumor volume and weight. Exosomes derived from H1975 cells can enhance the lncRNA H19 expression, and upregulate PTEN protein in H1975R cell xenograft tumor tissues, and enhance the anti-tumor efficacy of osimertinib. Taken together, targeting exosomal lncRNA H19 to upregulate PTEN and prevent the phosphorylation of AKT in H1975R cells may offer a promising therapeutic strategy against osimertinib resistance in H1975R cells.

However, the study has several limitations that remain unresolved. First, given that only a single paired cell line (H1975/H1975R) was utilized in this study, the extrapolation of our results might be limited. In addition, we did not perform gene mutation testing on the H1975R cells. Hence, future studies employing diverse cell lines are warranted to verify the biological functions and underlying mechanism of exosomal lncRNA H19, so as to make the research conclusions more reliable and universal.

## 5 Conclusion

**Figure 7:**
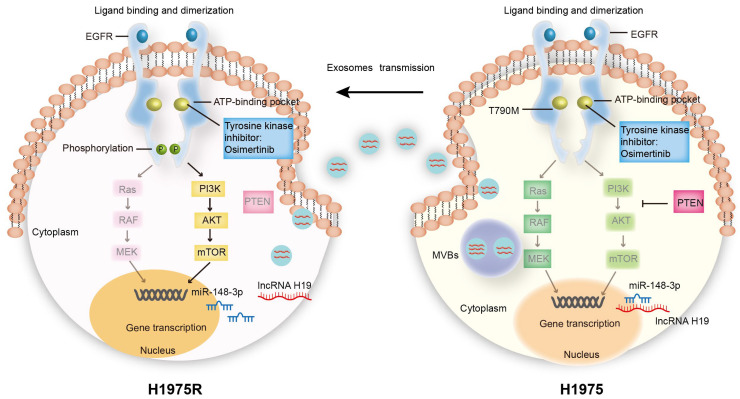
Schematic representation of the molecular mechanism underlying exosomal lncRNA H19-mediated reversal of osimertinib resistance. Exosomes transferred lncRNA H19 form H1975 cells to H1975R cells, and then upregulated the PTEN protein by sponging miR-148-3p, suppressing the activation of PI3K-AKT signaling in H1975 cells.

In conclusion, our findings demonstrate that exosomes carrying lncRNA H19 can reduce the miR-148-3p expression in osimertinib-resistant cells by the ceRNA mechanism and elevate the expression of the tumor suppressor PTEN (Fig. 7). PTEN plays important roles in negatively regulating PI3K-Akt signaling in NSCLC cells with EGFR-TKI resistance, the results also indicate that exosomes can efficiently transport antitumor bioactive substances to recipient cells, which may act as an efficient therapeutic tool for drug resistance. These results suggest a promising strategy to overcome osimertinib resistance.

## Data Availability

The data and materials supporting the results of this study are available from the corresponding author upon reasonable inquiry.
